# Spatial transmission of H5N2 highly pathogenic avian influenza between Minnesota poultry premises during the 2015 outbreak

**DOI:** 10.1371/journal.pone.0204262

**Published:** 2018-09-21

**Authors:** Peter J. Bonney, Sasidhar Malladi, Gert Jan Boender, J. Todd Weaver, Amos Ssematimba, David A. Halvorson, Carol J. Cardona

**Affiliations:** 1 Secure Food Systems Team, Department of Veterinary and Biomedical Sciences, University of Minnesota, Saint Paul, Minnesota, United States of America; 2 Department of Bacteriology and Epidemiology, Wageningen Bioveterinary Research, Wageningen University and Research Centre, Lelystad, The Netherlands; 3 Center for Epidemiology and Animal Health, Science Technology and Analysis Services, Veterinary Services, Animal and Plant Health Inspection Service, United States Department of Agriculture, Fort Collins, Colorado, United States of America; 4 Department of Mathematics, Faculty of Science, Gulu University, Gulu, Uganda; University of California Davis, UNITED STATES

## Abstract

The spatial spread of highly pathogenic avian influenza (HPAI) H5N2 during the 2015 outbreak in the U.S. state of Minnesota was analyzed through the estimation of a spatial transmission kernel, which quantifies the infection hazard an infectious premises poses to an uninfected premises some given distance away. Parameters were estimated using a maximum likelihood method for the entire outbreak as well as for two phases defined by the daily number of newly detected HPAI-positive premises. The results indicate both a strong dependence of the likelihood of transmission on distance and a significant distance-independent component of outbreak spread for the overall outbreak. The results further suggest that HPAI spread differed during the later phase of the outbreak. The estimated spatial transmission kernel was used to compare the Minnesota outbreak with previous HPAI outbreaks in the Netherlands and Italy to contextualize the Minnesota transmission kernel results and make additional inferences about HPAI transmission during the Minnesota outbreak. Lastly, the spatial transmission kernel was used to identify high risk areas for HPAI spread in Minnesota. Risk maps were also used to evaluate the potential impact of an early marketing strategy implemented by poultry producers in a county in Minnesota during the outbreak, with results providing evidence that the strategy was successful in reducing the potential for HPAI spread.

## Introduction

Beginning in January of 2015, the United States poultry industry experienced a severe outbreak of H5 highly pathogenic avian influenza (HPAI). Overall, HPAI was detected on commercial premises in nine states located primarily in the Midwest region of the United States [1]. The first detection of HPAI on a commercial premises occurred in California in January 2015, where a turkey premises had been infected with Eurasian HPAI H5N8 [2]. Shortly thereafter, Eurasian/American lineage HPAI H5N2 [3] was detected in commercial turkeys in the Midwest. HPAI spread was limited to no more than ten premises in seven of the nine states in which commercial premises were infected [1]. In Minnesota and Iowa, however, the outbreak was severe: 180 commercial premises were infected between these two states with devastating consequences [1]. The current study focuses on the state of Minnesota. The spatial spread of HPAI H5N2 during the 2015 outbreak in this state is analyzed through the estimation of a spatial transmission kernel. A spatial transmission kernel quantifies the infection hazard (likelihood of infection per a given unit of time, which in this analysis is a day) posed by an infectious premises to a susceptible premises located a given distance away.

Spatial transmission kernel approaches generally require only basic information and are especially useful when detailed between-premises contact data is limited or unavailable. For example, the transmission kernel model used in the current analysis is based only on infection status over time and inter-premises distance. The transmission kernel allows for broad inferences about the spatial scale of disease spread, and the relative importance of pathways characteristic of different inter-premises distances in causing infection. Furthermore, the spatial transmission kernel can be used in a number of applications for outbreak preparedness and emergency response planning. For example, a spatial transmission kernel in combination with data on premises location and length of the infectious period at the premises level can be used to estimate the basic reproduction number for each premises, which is the expected number of secondary infections a premises would cause if it was infectious while all other premises were susceptible. The basic reproduction numbers can in turn be used to formulate risk maps showing areas where a sustained outbreak could occur. Risk maps can be estimated under different hypothetical scenarios by varying the hazard rate or length of the infectious period of certain premises to approximate the effect of outbreak control strategies like culling or vaccination. Risk maps can then be compared to assess the strategy’s potential to limit outbreak spread. For examples see [4] or [5].

Spatial transmission kernels have been previously applied to model HPAI spread in a 2003 HPAI H7N7 outbreak in the Netherlands by Boender et al. [4], 1999–2000 HPAI H7N1 outbreak in Italy by Dorigatti et al. [6], and 1983–1984 HPAI H5N2 outbreak in the United States by Rorres et al. [7]. In the current analysis, a spatial transmission kernel model was used to: 1) provide insight into HPAI spread dynamics during the 2015 Minnesota outbreak, 2) identify areas with higher transmission risk in Minnesota, and 3) explore the impact of an early marketing outbreak management strategy implemented by turkey producers in one of the affected counties.

### HPAI H5N2 outbreak in Minnesota

**Fig 1:**
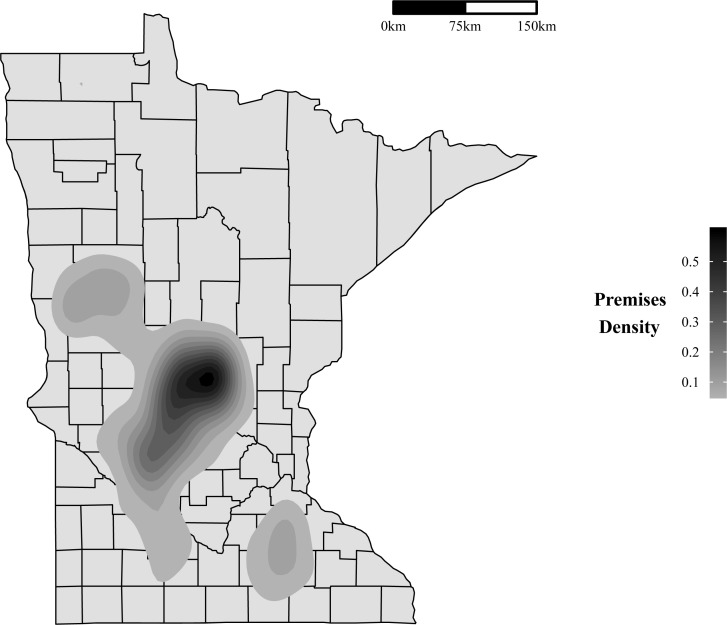
Density of poultry premises in Minnesota. Poultry density estimated from a bivariate normal kernel density [8].

The first HPAI-positive premises in Minnesota was officially diagnosed as H5 positive on March 4^th^, 2015. The outbreak lasted about three months, with the last case confirmed as H5 positive on June 4th, 2015. In total, 109 commercial poultry premises and 1 backyard flock were depopulated. These premises were primarily situated in the central part of the state, which has the highest density of commercial poultry premises. This can be seen in Fig 1, a density plot for poultry premises in Minnesota that was estimated using a bivariate normal kernel density [8]. Of the 109 depopulated commercial premises, 104 housed turkeys and five were egg-layer operations. One hundred and three of the commercial premises and the backyard flock were depopulated due to confirmed detection of HPAI. The 6 remaining depopulated commercial premises, all containing turkeys, were depopulated due to known dangerous contacts with an infected premises through shared equipment or personnel. These 6 dangerous contact premises were not included in the current analysis due to insufficient data. Thus, the case premises included in the analysis consist of 98 commercial turkey premises, 5 commercial egg layer premises, and 1 backyard flock.

Despite there being a substantial number of broiler producers in Minnesota, there were no detections of HPAI in broiler premises during the outbreak. An inoculation study of broilers with A/turkey/Minnesota/12582/2015 (H5N2) virus by Bertran et al. [9] found broilers to have lower susceptibility than layer chickens [10], but similar susceptibility as turkeys [11] to the virus. Therefore, Bertran et al. [9] hypothesized that the absence of infections in broiler premises during the outbreak were due to differences in production systems. In addition, contact structures conducive to the spread of infection could have been limited between the different poultry sectors, lowering the risk of infection posed by infected turkey and layer facilities to susceptible broiler premises due to fewer between-sector pathways.

**Fig 2:**
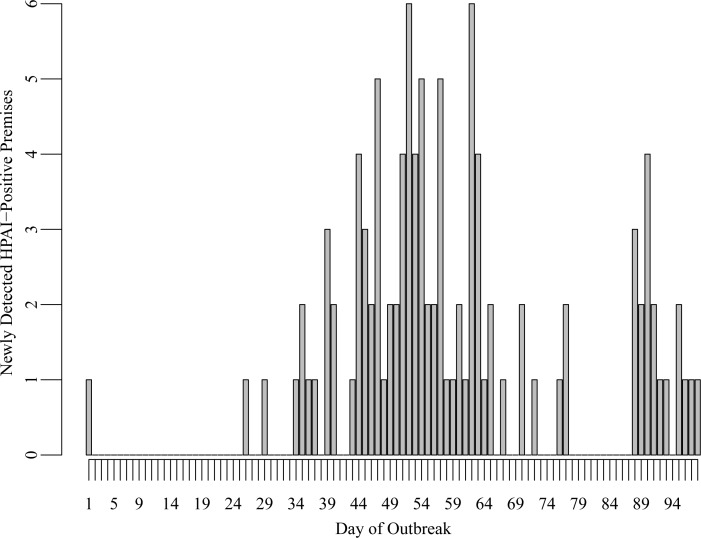
Number of premises newly detected with HPAI H5N2 per day during the 2015 outbreak in Minnesota. The absence of any new detections beginning the 78^th^ day and lasting through the 87^th^ day following the first detection was used to define two outbreak periods.

Fig 2 shows the number of premises newly detected with HPAI in Minnesota on each day of the outbreak. Notably there is a period of no new HPAI cases, occurring from the 78^th^ through the 87^th^ day following the first detection of HPAI. This period is preceded by a decreasing number of detections and followed by an increasing numbers of detections, which suggests the presence of two epidemic waves during the outbreak. Separate spatial transmission kernels were estimated for the first and second wave of detections to explore the possibility that some change in the outbreak conditions contributed to the increase in infections as part of the second wave.

### HPAI control measures in Minnesota

Infected premises were immediately quarantined, with depopulation and disposal of carcasses as soon as resources were available. Live bird and poultry product movements were restricted for uninfected premises located within a Control Area with a radius of 10 km established around each infected premises [12]. Movement within, into, and out of the Control Area was governed by a permitting system requiring the implementation of strict biosecurity measures in combination with active surveillance diagnostic testing and monitoring of daily mortality in the flock, used here to refer to all the birds housed on the premises. The Control Area consisted of two zones, the Infected Zone and Buffer Zone. The Infected Zone was comprised of the area within a radius of at least 3 km surrounding the infected premises. The Buffer Zone was comprised of the area from the border of the Infected Zone to the border of the Control Area. Premises located outside of the Control Area (i.e., the Free Area) performed routine surveillance. Control Areas were released twenty-one days after the last infected premises had begun cleaning and disinfection activities with no new cases of HPAI having occurred. These control measures remained unchanged throughout the outbreak period.

### Early marketing outbreak control strategy

Early marketing refers here to the practice of sending houses to processing earlier than the normal scheduled date during an outbreak in order to reduce the density of commercial poultry in a given area. Therefore, early marketing is similar to preemptive culling methods like those discussed in Boender et al. [4] in that both strategies aim to reduce the potential for outbreak spread by reducing the density of susceptible poultry. Preemptive culling traditionally consists of depopulating all premises within a certain distance from an infected premises as soon as possible. The early marketing strategy, on the other hand, involves producers subjectively selecting premises for depopulation over time. The decision to market is informed by the perception of the infection risk for each premises and may be based on relevant factors such as the age of the birds housed on the premises and/or density of premises in the area. Early marketing normally leads to partial reduction in the total number of premises with live birds in the region surrounding an infected premises. The strategy aims to be less economically disruptive than traditional preemptive culling methods while still effectively reducing the potential for outbreak spread by managing poultry density.

## Materials and methods

### Outbreak data

Data used to estimate the spatial transmission kernel consisted of all poultry premises located in the U.S. state of Minnesota during the 2015 HPAI H5N2 outbreak. It contained the locations, used to calculate the inter-premises distances, of 104 premises that were confirmed HPAI-positive and 662 premises that remained uninfected during the outbreak period. Six premises that were treated as infected and depopulated due to known dangerous contacts with an infected premises through shared equipment or personnel were excluded from the analysis, since their depopulation dates were not reported. For each case premises, the date on which HPAI was first detected through diagnostic testing, heightened mortality, or the observation of clinical signs were provided. In addition, the scheduled disposal dates of the depopulated poultry carcasses, performed via composting or burial, were provided for 96 of the infected premises. For the eight premises whose disposal start dates were missing, the dates were estimated using the average number of days between detection and disposal of the case premises with complete information. The uninfected premises data consisted of 37 broiler breeders, 176 broiler meat-type premises, 22 egg layers, 13 pullet farms, 2 chicken hatcheries, 6 turkey hatcheries, 64 turkey breeders, and 342 turkey meat-type premises. The only backyard flock included was the single confirmed HPAI-positive backyard flock.

### Early turkey marketing data

A second dataset from a county in Minnesota in which poultry producers performed early marketing of turkeys was obtained. There were 50 total poultry premises in this county, 14 of which, all turkey premises, performed early marketing of at least one house. There is some uncertainty concerning how many of the 14 premises performed early marketing while in a Control Area, as data on the date each Control Area was established was not available for this study. For an approximate estimate, of the 14 premises that performed early marketing, 11 were within 10 km (the size of a Control Area) of at least one confirmed infected premises at the time of early marketing. All houses that were early marketed while in a Control Area tested negative for avian influenza prior to movement of birds to processing. The early marketing data contained the age of the birds in the house that was marketed early, the date on which the early marketing occurred, and the date on which the house would have been marketed regularly. The ages of the other birds housed on the premises were also indicated. For those turkey premises that did not send a house to processing early and were never infected during the outbreak, the ages of all birds housed on the premises on April 1^st^, 2015 were provided as well as the marketing date. The ages of birds housed on case premises were given at the time of detection. Age and marketing information were not provided for the premises that did not house turkeys and were never infected during the outbreak.

### Spatial transmission kernel model

In this study, four transmission kernel formulations evaluated by Hayama et al. [13] in an analysis of the 2010 foot and mouth disease (FMD) outbreak in Japan were assessed for best fit. These formulations are derived from studies by Boender et al. [4], Boender et al. [5], and Chis Ster et al. [14] analyzing disease spread during outbreaks of HPAI H7N7 in the Netherlands in 2003, FMD in the Netherlands in 2001, and FMD in Great Britain in 2001, respectively. The four formulations are given below with the hazard rate (h) as a function of *d*_*ij*_, the distance in kilometers between infectious premises *i* and uninfected premises *j*. The three parameters *h*_0_, *r*_0_, and *α* are unknown constants that must be estimated from outbreak data. These formulations are later referred to as Models 1 through 4:

$$h(d_{ij})=\frac{h_{0}}{1+{\left(\frac{d_{ij}}{r_{0}}\right)}^{\alpha }}$$ 1.

$$h(d_{ij})=h_{0}\left(1-exp\;(-{\left(\frac{d_{ij}}{r_{0}}\right)}^{-\alpha })\right)$$ 2.

$$h(d_{ij})=h_{0}exp\;\left(-{\left(\frac{d_{ij}}{r_{0}}\right)}^{\alpha }\right)$$ 3.

$$h(d_{ij})=h_{0}{\left(1+\frac{d_{ij}}{r_{0}}\right)}^{-\alpha }$$ 4.

All these formulations depend exclusively on inter-premises distance and infection status over time. Other factors such as premises type are not captured. Model 1 was considered the baseline model due to its use in analyses of two previous HPAI outbreaks, the 2003 HPAI H7N7 outbreak in the Netherlands and 1999–2000 HPAI H7N1 outbreak in Italy [4, 6]. In the baseline model, the *h*_0_ parameter represents the maximum hazard rate, occurring when *r*_0_ = 0. The *r*_0_ parameter influences how far the hazard rate extends over distance, and the *α* parameter controls the rate of decline in the hazard rate from the maximum.

Here, the transmission kernel parameters were estimated using a maximum likelihood approach as described in [4]. Infection status was defined at the premises level and had the categories susceptible, infected (the time when the premises first contracted the infection), infectious, and removed. The infection status was updated by daily increments. Inter-premises Euclidean distances were used in the estimation of the kernel parameters [15].

To derive the likelihood function, the following expressions must be defined. Taking *λ*_*i*_(*t*) as the cumulative hazard rate experienced by premises *i* on day *t*, termed the force of infection, the probability that premises *i* is infected on day *t* is

$$q_{i}(t)=1-e^{-\lambda_{i}(t)},$$

and the probability that premises *i* remains uninfected up to day *t* is

$$r_{i}(t)=e^{-\sum_{s=1}^{t-1}\lambda_{i}(s)}.$$

As given in [4], the force of infection is defined by the following expression,

$$\lambda_{i}(t)=\sum_{i\neq j}h(d_{ij})1\{j\mathrm{is}\;\mathrm{infectious}\},$$

Based on the above equation, the force of infection experienced by susceptible premises *i* on day *t* depends on the total number of infectious premises on day *t*, and the distances between the infectious premises and premises *i*. If there are no infectious premises on day *t*, the force of infection is zero. However, a phylogenetic analysis conducted by the United States Department of Agriculture (USDA) found evidence that, during this outbreak, primary introductions occurred concurrently with lateral spread in the Midwest [16]. This result indicates the presence of an infection risk that is independent of the risk posed by infectious premises. To allow for the possibility for a susceptible premises to become infected through a primary introduction, an additional parameter *k* was introduced into the force of infection, resulting in the following expression,

$$\lambda_{i}(t)=\left(\sum_{i\neq j}h(d_{ij})1\{j\mathrm{is}\;\mathrm{infectious}\}\right)+k.$$

As introduced, the *k* parameter represents a constant infection risk on day *t*: The risk contributed to the force of infection by *k* does not change based on the number of infectious premises on day *t* or the between-premises distances. During parameter fitting *k* may capture some risk posed by long distance movements of people and equipment. Even though the cumulative risk posed by these movements would be expected to vary with the number of infectious premises, some of the risk could still be captured by *k* due to long distance movements resembling distance independent transmission. To confirm *k* is behaving as intended, a fifth parameter, δ, was introduced into the model. While the addition of δ did not end up improving the model fit, it still allowed for the performance of *k* to be evaluated. The δ parameter was incorporated into the force of infection as follows,

$$\lambda_{i}(t)=\left(\sum_{i\neq j}\left(h(d_{ij})+\delta )1\{j\mathrm{is}\;\mathrm{infectious}\right\}\right)+k.$$

δ is a distance-independent parameter whose total contribution to the force of infection is proportional to the total number of infectious premises. Therefore, δ should capture the risk posed by the long distance movements presumed to resemble distance independent transmission, as this risk should still depend on the number of infectious premises. Since these are the movements that could possibly confound *k*, it is expected that the addition of the δ parameter reduces the likelihood of the *k* parameter estimate being confounded.

The comparative fits for the different formulations of *λ*_*i*_(*t*) to the outbreak data were evaluated using Akaike’s Information Criterion (AIC) [17]. In this criterion, two models with a difference in AIC of less than two are not considered to be significantly different in terms of fit to the data, while a difference of greater than ten is strong evidence that the model with the smaller AIC is a better fit [18].

Let *K* be the set of premises that were never infected during the outbreak, Ʌ be the set of premises culled on day *t*_*cul*,*l*_, *M* be the set of premises that were infected on day *t*_*inf*,*m*_, and *t*_*max*_ be the final day of the outbreak. Then the likelihood function for the estimation of the parameters is given by

$$L=\prod_{k\in K}r_{k}\left(t_{max}\right)\prod_{l\in Ʌ}r_{l}(t_{cul,l})\prod_{m\in M}r_{m}(t_{inf,m})q_{m}(t_{inf,m}).$$

In order to estimate the transmission kernel parameters, the log of the likelihood function L was optimized via the “nlminb” algorithm, a bounds constrained quasi-Newton method in R’s “optimx” function [19, 20]. In the optimization procedure, the parameter space was bounded as follows, *h*_0_ ∈ [0.0,0.1], *r*_0_ ≥ 0, *α* ≥ 0, *k* ≥ 0, and *δ* ≥ 0, and the confidence intervals for the parameters were estimated using the profile likelihood method. Throughout the analysis, data was processed and analyzed using R statistical software, in particular the “optimx” and “ggsn” packages, and Mathematica [19–23].

### Model scenarios

Under the baseline scenario, which includes all premises in Minnesota, a case premises was assumed to have been infected eight days prior to the reported detection date. Upon infection, the premises was assumed to be latently infected for three days. The infectious period starts following the three-day latent period and lasts until the scheduled start date of carcass disposal following depopulation. The eight days to detection, which includes the three day latent period, were set based on results from a simulation study involving re-parameterization of the within-house turkey disease transmission model described by Weaver et al. [24] for the Minnesota HPAI H5N2 strain. A median time to detection following HPAI exposure of 8 days was estimated from the simulation model under a surveillance program consisting of a mortality trigger of 2 birds per 1000, which would be representative of the surveillance program performed by premises outside of a Control Area. Since diagnostic testing performed by premises within a Control Area would reduce the time to detection, the 8 days used in the transmission kernel analysis is a more conservative estimate. The three day latent period was established based on the finding that very few infections were predicted to occur in the first three days following the initial introduction. Clearly, there is uncertainty surrounding the time of infection and transitions in infectious status for each infected premises, and the deterministic approach used here is a limitation of this study. However, the approach used is similar to that of [4] and [6], and a sensitivity analysis performed suggested that the transmission kernel parameter estimates are robust to changes to the definitions regarding the transitions in infection status.

Transmission kernel parameters were estimated under the baseline scenario for three outbreak periods: the entire outbreak, which was defined to begin on the day the first case premises was estimated to have been infected and end after the carcass disposal date of the last infected premises, as well as a first and second wave defined based on the daily number of newly detected HPAI-positive premises given in Fig 2. The first wave was defined to begin on the day the first case premises was estimated to have been infected and last through the 81^st^ day following the earliest detection date. The second wave was defined to begin on the 82^nd^ day following the first detection date and end following the carcass disposal date of the last infected premises.

Since no broiler premises were infected during the outbreak, a variant of the baseline scenario was evaluated in which broiler breeder and meat-type chicken premises were excluded from the analysis. This second scenario was motivated by the hypothesis that broiler premises may have had low susceptibility due to inherent differences in the production system that reduced the likelihood of exposure and/or minimal epidemiological contact between poultry sectors, which would have led to broiler premises having limited contact with infectious premises.

To assess the effect of the uncertainty regarding the day of infection and length of the infectious period, the baseline scenario was adjusted to observe the potential impact of extended infectiousness beyond the start of carcass disposal. For this assessment, a third scenario in which the infectious period was assumed to last through the fifth day following the disposal start date was evaluated. This extension was set primarily based on the relationship between the detection date and carcass disposal start date in the data to ensure that there was an overlap of the infectious period of the first case and the assumed date of infection of the second case of the outbreak. However, the assumption is not unreasonable, as HPAI virus has been found to persist in substrates such as feces for considerable amounts of time. For example, in a laboratory setting at 4 degrees Celsius, Wood et al. [25] found virus in chicken feces after 13 days and Beard et al. [26] found virus in chicken feces after 35 days. Therefore, it is possible for infectiousness at the premises level to have continued after the disposal start date prior to cleaning and disinfection.

### Risk maps based on basic reproduction numbers estimated from the spatial transmission kernel

A premises’ basic reproduction number is the expected number of premises it would infect if all other premises were susceptible. When basic reproduction numbers are greater than one, there is a non-zero probability of an epidemic, while basic reproduction numbers less than one signify sustained spread would not occur. Therefore, basic reproduction numbers can be used to create risk maps showing the areas where sustained spread is possible by indicating which premises have a reproduction number larger than one.

The basic reproduction number was estimated for each farm in Minnesota following the approach in Boender et al. [4], which utilizes the spatial transmission kernel. Let ${\tilde{T}}_{i}$ be the length of the stochastic infectious period of premises *i*, and *j* be the index identifying another premises. The reproduction number of premises *i*, *R*_*i*_, is given by

$$R_{i}=\sum_{j\neq i}\left(1-E[e^{-h(d_{ij}){\tilde{T}}_{i}}]\right).$$

The stochastic infectious period, ${\tilde{T}}_{i}$, was estimated by fitting a gamma distribution to the lengths of the infectious periods observed in case premises during the Minnesota outbreak under the baseline assumptions regarding infection status. In order to more closely mimic the transmission risk as it was during the 2015 Minnesota outbreak, i.e., involving no broilers, the mean parameter estimates under the scenario excluding broilers were used to estimate the reproduction numbers for non-broiler premises only.

### Early marketing risk map example

The transmission kernel based risk maps have previously been used to predict the effectiveness of different outbreak control strategies such as culling or vaccination in reducing the potential for the spread of infection. For examples see [4] and [5]. In the current study, risk maps were used to explore the effects of early marketing on the disease dynamics based on the data from the county in Minnesota in which poultry producers performed early marketing. For this analysis, risk maps were generated for a selected date considering the at-risk premises with and without the early marketing having been performed. At-risk premises were defined as non-broiler premises that had not been infected on or before the selected date and turkey premises with at least one house that contained birds nine weeks old or older. Turkey premises with birds solely younger than nine weeks old were excluded from the at-risk designation. Turkeys less than nine weeks old were assumed not to be susceptible based on the September 2015 USDA Epidemiological report which concluded that changes in susceptibility due to age (specifically using 9 weeks as reference age) or farm practices associated with certain ages could have increased the risk of infection as birds grew older [16].

The date chosen for the risk maps was April 14^th^, 2015. On this date seven of the 14 early market premises had sent at least one house to processing and 11 of the 14 case premises located in the preemptive marketing county had been infected out of a county-wide total of 50 non-broiler premises. Thus, the date provides a reasonable snapshot of the infection risk while flocks were being marketed down, as a nontrivial number of houses had been processed prior to the regular market date even as the infection continued to move through the county. Two risk maps were generated for this date, one that considers when premises were sent early to market and, conversely, one that considers when these premises would have marketed regularly. The no early marketing scenario is a hypothetical scenario designed to observe the potential effect of an increased number of susceptible premises in the county due to the greater number of older turkeys present waiting to be marketed.

## Results

### Model comparisons

Due to the phylogenetic evidence suggesting its use, the four transmission kernel formulations compared included the *k* parameter in the force of infection during parameter estimation. None of the negative log-likelihoods (-LL) of the four formulations differed by more than two (-LL = 1385.393 for Model 1, -LL = 1385.392 for Model 2, -LL = 1384.030 for Model 3, and–LL = 1384.067 for Model 4), which suggests that none of the formulations were definitively the best fit. Under this uncertainty, Model 1 was chosen for the current analysis due to its use by Boender et al. [4] and Dorigatti et al. [6] in analyses of HPAI outbreaks in the Netherlands and Italy, respectively.

Since the second case was infected on a day when there were no infectious premises under the baseline assumptions, the model was undefined when the *k* parameter was not included. However, when the first case, as a possible isolated introduction, was excluded from the model fitting, the model with *k* continued to perform better (AIC = 1353.379 with *k* and AIC = 1383.895 without *k*). Furthermore, when all premises were included under the extended infectious period scenario, the model with *k* had AIC equal to 1406.329 while the model without *k* had AIC equal to 1434.345. Having identified *k* as significantly improving the model fit, the relative contribution of long distance transmission related to infectious premises to the infection risks represented by *k* was assessed using the parameter δ. When δ is included in the model, the AIC was equal to 1395.332 as compared to the AIC of 1393.393 without δ, so the addition of δ did not significantly improve the model fit. In addition, the maximum likelihood estimate for *k* remained nearly unchanged with the inclusion of δ (*k* = 0.00031 with *δ* and *k* = 0.00032 without *δ* under the baseline scenario assumptions).

### Spatial transmission kernel parameter estimates

The spatial transmission kernel parameter mean estimates and 95% confidence intervals for the 2015 Minnesota HPAI H5N2 outbreak scenarios are given in Table 1. Under the baseline estimate for *k* given in Table 1, the daily probability of a premises becoming infected through a pathway associated with *k*, namely distance independent pathways largely unrelated to the number of infectious premises, is estimated to be 0.032%. Over 115 days, the length of the Minnesota outbreak under the baseline scenario, approximately 28 (95% CI: 14–46) out of 766 susceptible premises would be expected to become infected through one of these pathways. The model parameters do not differ significantly between the baseline, no broilers, and extended infectious period scenarios. However, in the split outbreak scenario, the parameter estimates for *r*_0_ and *k* in the first and second wave do differ significantly.

**Table 1:** Minnesota spatial transmission kernel maximum likelihood estimates.

Scenario	*h*_0_	*r*_0_	*α*	*k*
Baseline	0.0061 (0.0025, 0.0137)	7.02 (3.07, 16.16)	2.46 (1.80, 4.38)	0.00032 (0.00016, 0.00052)
First Wave	0.0078 (0.0035, 0.0179)	5.50 (2.29, 11.56)	2.37 (1.72, 3.66)	0.00043 (0.00022, 0.00068)
Second Wave	0.0017 (0.0007, 0.0053)	30.51 (18.88, 41.81)	14,623.59 (3.16, Inf)	0.00 (0.00, 0.00017)
Broilers Excluded	0.0056 (0.0023, 0.0140)	7.18 (2.58, 17.08)	2.13 (1.52, 4.00)	0.00042 (0.00017, 0.00072)
Extended Infectious Period	0.0042 (0.0011, 0.0098)	8.18 (3.49, 26.48)	2.75 (1.93, 14.30)	0.00034 (0.00018, 0.00052)

Mean spatial transmission kernel parameter estimates with 95% confidence intervals (in parenthesis) estimated from the 2015 HPAI H5N2 outbreak in Minnesota for each scenario and outbreak period. The ***h***_**0**_ parameter represents the maximum hazard rate, while ***r***_***0***_ and ***α*** together determine the rate of decline from the maximum and the magnitudes of distance over which the decline occurs. The ***k*** parameter represents a distance independent infection hazard posed primarily by transmission pathways independent of the number of infectious premises.

**Fig 3:**
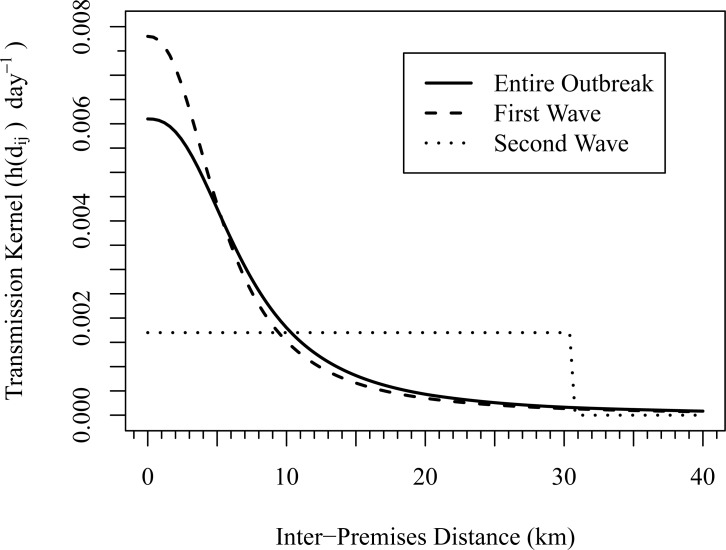
Minnesota spatial transmission kernels. Spatial transmission kernels estimated from the three different outbreak periods evaluated across distance using the mean parameter estimates under the baseline scenario given in Table 1.

Fig 3 shows a plot of the hazard rate over distance as estimated by the mean spatial transmission kernel estimates for the first wave, second wave, and entire outbreak. Under the baseline scenario, the entire outbreak had a length of 115 days, the first wave had a length of 90 days, and the second wave had a length of 25 days. Furthermore, 86 premises were detected during the first wave period, while 18 premises were detected during the second wave period. The mean estimates clearly illustrate differing behavior in the between-premises transmission between the two waves. Uniform transmission risk within 30 km was estimated from the second wave cases, whereas the transmission risk was estimated to decline steadily over distance in the first wave, falling below the constant risk of the second wave after about 10 km.

Table 2 provides spatial transmission kernel parameter estimates from two additional outbreaks, the HPAI H7N7 outbreak in the Netherlands in 2003 and HPAI H7N1 outbreak in Italy in 1999–2000. The 2003 HPAI H7N7 Netherlands outbreak transmission kernel was estimated by Boender et al. [4] under their default scenario, which assumes case premises become infected six days prior to the first rise in mortality and that the infectious period spans from two days after infection until depopulation. Similarly, the 1999–2000 HPAI H7N1 Italy outbreak transmission kernel shown was estimated by Dorigatti et al. [6] under their basic model scenario, which assumes case premises become infected seven days prior to detection, with the infectious period starting two days after infection and ending once the case premises has been depopulated. Boeder et al. [4] and Dorigatti et al. [6] estimated their respective transmission kernels using the Model 1 parameterization. However, the outbreaks in both studies were considered to have involved a single primary introduction, so an additional *k* parameter was not added to the force of infection.

**Table 2:** Comparison of spatial transmission kernel estimates from Minnesota, the Netherlands, and Italy.

HPAI Outbreak	*h*_0_	*r*_0_	*α*	*k*
Minnesota 2015HPAI H5N2	0.0061 (0.0025, 0.0137)	7.02 (3.07, 16.16)	2.46 (1.80, 4.38)	0.00032 (0.00016, 0.00052)
Netherlands 2003HPAI H7N7(Estimates from Boender et al. [4])	0.0020 (0.0012, 0.0039)	1.9 (1.1, 2.9)	2.1 (1.8, 2.4)	NA
Italy 1999–2000 HPAI H7N1 (Estimates from Dorigatti et al. [6])	0.0064 (0.0037, 0.0090)	2.15 (1.39, 2.91)	2.08 (1.87, 2.28)	NA

Mean parameter estimates and 95% confidence intervals (in parenthesis) estimated from HPAI outbreaks in Minnesota under the baseline scenario, the Netherlands by Boender et al. [4], and Italy by Dorigatti et al. [6], all using the Model 1 parameterization of the spatial transmission kernel.

**Fig 4:**
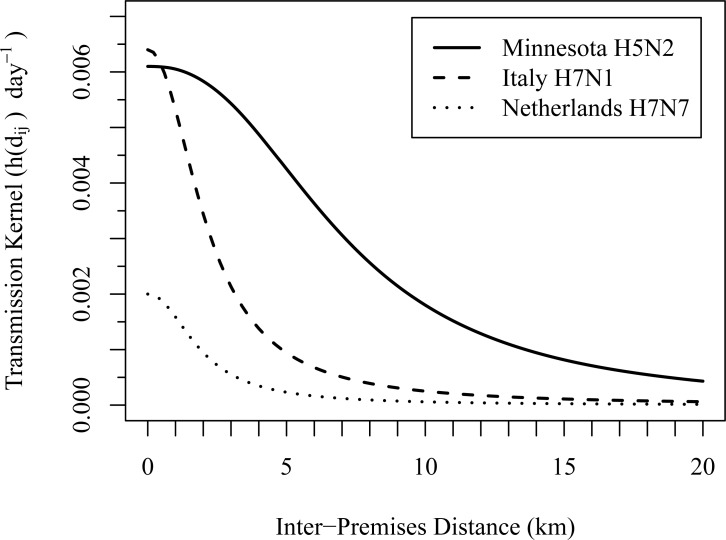
Minnesota, Italy, and Netherlands spatial transmission kernel comparison. Spatial transmission kernels evaluated across distance using the mean parameters estimated from the 2015 Minnesota HPAI H5N2 outbreak under the baseline scenario, the 1999–2000 Italy HPAI H7N1 outbreak under the Dorigatti, Mulatti (6) basic model scenario, and the 2003 Netherlands HPAI H7N7 outbreak under the Boender, Hagenaars (4) default scenario. Table 2 for the Minnesota, Dorigatti, Mulatti (6), and Boender, Hagenaars (4) transmission kernel estimates.

The transmission kernel parameter estimates from Boender et al. [4] and Dorigatti et al. [6] differ significantly from the baseline Minnesota estimates only in the *r*_0_ parameter, which suggests between-premises transmission occurred over longer distances in the Minnesota outbreak. Fig 4 plots the mean spatial transmission kernels from the estimates given in Table 2 and the 2015 HPAI H5N2 Minnesota outbreak estimates under the baseline scenario. From Fig 4 it is clear that the mean transmission risk was primarily local in the HPAI outbreaks in the Netherlands and Italy. Under the Minnesota kernel, however, due to the significantly larger estimated *r*_0_, infectious premises continue to pose a nontrivial infection risk to susceptible premises at moderate distances.

### Minnesota outbreak risk maps

**Fig 5:**
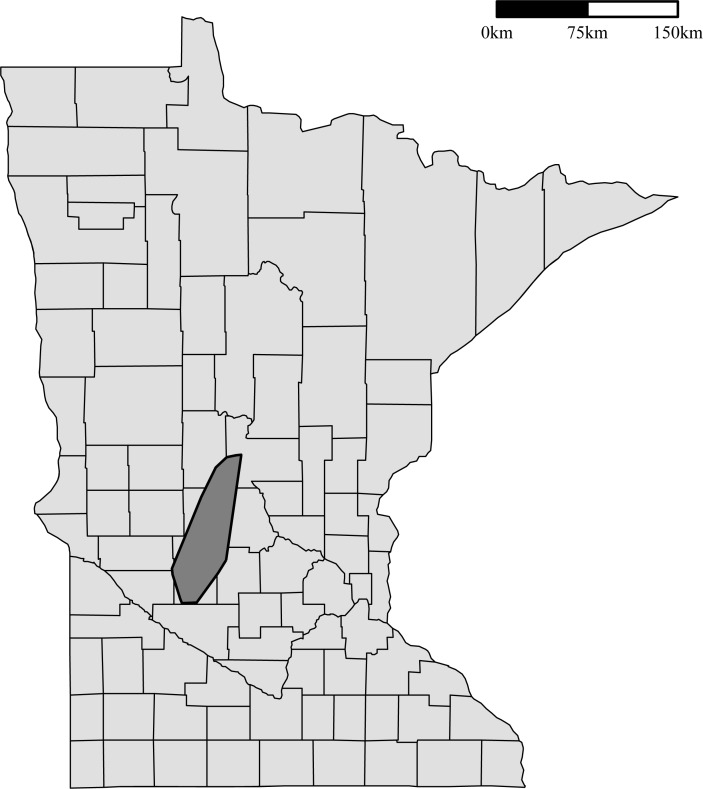
Minnesota risk map. The shaded region encapsulates the primary risk area for HPAI spread in Minnesota with broiler type premises excluded. The risk area was identified by the premises with basic reproduction numbers greater than one. See text for details.

The shaded area in Fig 5 contains the high risk area in Minnesota where premises have reproduction numbers larger than one, which means an infection within this area may result in a sustained outbreak. During the outbreak, 57 of the 184 (31%) premises (not including broilers) in the high risk area were infected, while 47 of the 381 (12%) premises outside of the high risk area were infected. Broken down by the two waves, 51 case premises in the high risk area and 35 case premises outside of the high risk area were detected during the first wave, while 6 case premises in the high risk area and 12 case premises outside of the high risk area were detected during the second wave. Furthermore, premises detected with HPAI outside of the high risk area during the first wave were dispersed throughout Minnesota, whereas the premises detected with HPAI outside of the high risk area during the second wave formed a linear cluster in the south-central part of the state. Risk maps generated from the transmission kernels estimated from the first and second wave periods did not differ substantially from the map produced by the transmission kernel estimated from the overall outbreak.

**Fig 6:**
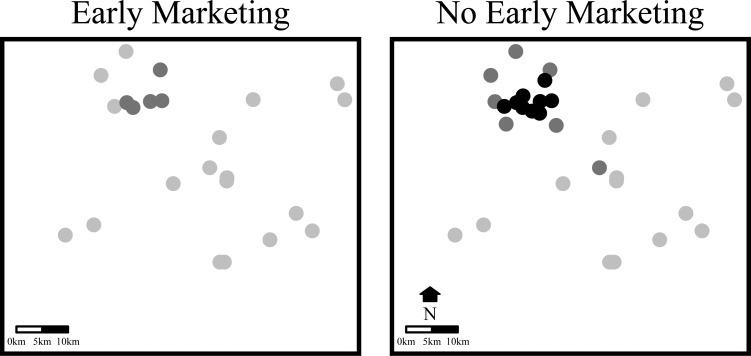
Controlled marketing risk map. Risk maps considering the number of susceptible premises on April 14^th^, 2015 in a county in Minnesota that sent houses early to processing prior to the regular market date as an outbreak response measure. The plot to the left was estimated considering the susceptible premises with early marketing having been performed, while the plot to the right was estimated assuming houses had been marketed regularly. Light gray points are low risk premises (***R***_**0**_ **< 0.7**), dark gray points are moderate risk premises (**0.7 ≤ *R***_**0**_ **≤ 1.0**), and black points are high risk premises (**1.0 < *R***_**0**_).

Fig 6 contains risk maps for the early marketing county on April 14^th^, 2015 considering those premises defined as susceptible with and without the early marketing strategy having been implemented. The risk maps show a close-up of all poultry premises in the county. The square boundaries of the plots do not represent the county boundaries, which were not displayed in order to preserve anonymity. The light gray points are premises with basic reproduction numbers less than 0.7, meaning the risk of these premises sustaining infection spread is low. Dark gray points are medium risk premises with basic reproduction numbers greater than or equal to 0.7 and less than or equal to 1.0, and black points are high risk premises, which have basic reproduction numbers greater than 1.0. On this date, there was estimated to have been no high risk, five moderate, and 18 low risk premises following the implementation of early marketing. When early marketing is assumed to not have occurred, there was estimated to have been nine high risk, seven moderate, and 14 low risk premises. The seven additional premises in the “no early marketing” scenario are the turkey premises with birds aged older than 9 weeks awaiting processing at the regularly scheduled date. These additional susceptible premises sufficiently increase the density to create a high risk area within the county.

## Discussion

In this study, the spread of HPAI H5N2 in Minnesota during the 2015 outbreak was analyzed using a spatial transmission kernel. The mean infection hazard from the transmission kernel for a susceptible premises located some given distance from an infectious premises theoretically averages the risk over all possible transmission pathways at that distance. An individual premises’ risk of becoming infected could be higher or lower than that predicted by the transmission kernel depending on the premises’ specific contact structure network. The spatial transmission kernel is a summary estimate that can provide insight into the relative infection risk posed by the typical pathways at different distances and the scale of the spread during the outbreak.

The transmission kernel parameter estimates given in Table 1 for the baseline, no broilers, and extended infectious period scenarios indicate no significant difference. This suggests that the estimates are robust to changes in the assumptions regarding infection status and that the broiler premises did not impact outbreak spread behavior, which could be due to the location of broiler premises compared to infected premises. On average, the closest case premises to a broiler premises was 41.97 km away. For comparison, the closest case premises to a turkey premises that was never infected during the outbreak was on average 27.03 km away. Similarly, there were 23 broiler premises (11.11%) within 10 km (the Control Area size) of at least one case premises, while the total number of never-infected turkey premises within 10 km of a case premises was 108 (28%). Both of these numbers suggest that broiler premises were, in general, located relatively far from the infected premises in the outbreak. Since the transmission kernel is distance dependent, this could have resulted in the broiler premises having a limited effect on the transmission kernel parameter estimates.

The baseline parameter estimates indicate spread of HPAI was primarily distance dependent during the Minnesota outbreak. Though there is a significant distance-independent transmission risk depicted by *k*, the distance-dependent risk of the transmission kernel is relatively greater. Under the baseline scenario, transmission related to *k* would be expected to result in 28 infections based on the number of premises at the start of and on the length of the outbreak in Minnesota. This would account for less than a third of the 104 infected premises.

As defined, *k* is independent of both distance and the number of infectious premises. Even though *k* does not depend on the number of infectious premises, it could potentially have captured some of the risk related to long distance movements of people and equipment due to some of these movements resembling distance independent pathways. Because the addition of the δ parameter, which was defined to be distance independent but dependent on the number of infectious premises, did not significantly improve the model, as demonstrated by the AIC value, the effect of long distance movements on *k* was likely small. Thus, the majority of the 28 infections attributed to *k* could be primary introductions of HPAI virus from environmental pathways. These environmental pathways are likely directly related to wild birds, as wild birds could spread virus in a manner consistent with the definition of *k*, i.e., independent of distance and the number of infectious premises. Indeed, wild waterfowl have often been suspected of causing the initial introduction into commercial poultry in previous outbreaks [27], and are suggested by Garber et al. [28] as the likely source of initial introduction and spread into new areas during the 2015 HPAI outbreak in the United States. Ideally, since the spatial transmission kernel assumes infections are due to lateral spread, separate transmission kernels should be fit to each introduction and resulting infections as identified through a method such as phylogenetic clustering or mapping of epidemiological links. Lacking such data, *k* is used as a complementary method, although still represents a less ideal approach.

Based on the transmission kernel results, lateral spread was distance dependent, with transmission risk the highest at close distances to an infectious premises. For example, the daily infection hazard posed by an infectious premises to a susceptible premises located 2 km away, which would place the susceptible premises in the center of a standard Infected Zone, was estimated to have been 0.0058, while the daily infection hazard posed by an infectious premises to a susceptible premises located 7 km away, which would be in the center of a standard Buffer Zone, was estimated to have been 0.0031. The reduction in infection risk from the center of a standard Infected Zone to the center of a standard Buffer Zone supports the assertion that distance-based control measures can be an effective strategy to manage the risk of infection spread. However, the transmission kernel estimates the hazard rate to have remained sizeable over an extended distance, even at distances exceeding the 10 km border of the Control Area. The daily infection hazard posed by an infectious premises to a susceptible premises located 11 km away, for example, was estimated to have been 0.0015. Therefore, while the Control Area can be an effective risk management tool, tracing dangerous contacts between premises is also of critical importance in managing outbreak spread. The distance dependent transmission kernel results suggest that local transmission pathways such as equipment sharing, or movement of wild animals (e.g., raccoons or foxes) or people between premises [29, 30] contributed substantially to outbreak spread. However, the sizeable hazard rates estimated for extended distances from an infectious premises are evidence that contact occurring over moderate distances, for example garbage and/or rendering truck visits to multiple premises, in addition to the local contacts, may have contributed to between-premises HPAI spread during the Minnesota outbreak [28].

A comparison of the parameters estimated from the first and second wave in Table 1 reveals that the two outbreak periods differ significantly in the *r*_0_ and *k* parameters, which is evidence of a change in outbreak spread during the second wave period. Based on the parameter estimates from the second wave, transmission of HPAI appears to have been from distance independent lateral spread occurring over the roughly 30 km surrounding an infectious premises. The estimates for the first wave, on the other hand, are similar to the estimates for the overall outbreak. This result is unsurprising since 83% of the case premises were detected during the first wave. As for the overall outbreak, *k* is estimated to be significantly greater than zero, which is evidence of distance-independent transmission, likely from environmental hazards, occurring during the first wave. However, distance-dependent lateral spread, as represented by the transmission kernel, is estimated to have posed a relatively greater risk.

Based on the differences in the transmission kernels estimated from the two outbreak periods, distance independent lateral spread likely played a larger role in HPAI transmission during the second wave relative to the first wave. The linear clustering of the case premises outside of the high risk area in the second wave is suggestive of a shared transmission mechanism associated with a roadway, such as if the premises were along a major rendering route or common route for transporting birds to slaughter. These transmission mechanisms could easily occur over long distances and lead to distance independent spread as observed in the second wave transmission kernel. As the clustered premises outside the risk area defined by the spatial kernel comprise two thirds of the total number of premises infected during the second wave, the relative contributions of roadway-based risks to HPAI spread during the second wave as compared to the first wave could result in different parameter estimates.

A comparison of spatial transmission kernels from different outbreaks is not straightforward due to the complexity of differences between outbreaks and summary nature of the spatial transmission kernel. However, such a comparison can suggest how certain industry structures or practices influenced spread during a particular outbreak. Here, the spatial transmission kernel parameters estimated from a 2003 HPAI H7N7 outbreak in the Netherlands and 1999–2000 HPAI H7N1 outbreak in Italy are compared with the baseline estimates from the 2015 HPAI H5N2 outbreak in Minnesota, all given in Table 2.

The *r*_0_ parameters estimated from the European outbreaks differ significantly from the *r*_0_ estimate from the Minnesota outbreak under the baseline scenario, with the results suggesting between-premises transmission occurred over longer distances in the Minnesota outbreak. Given the magnitude of the *r*_0_ estimate from the Minnesota outbreak, the transmission at longer distances is likely a result of differences in the activities or behaviors related to movement of people and equipment. It is possible that Minnesota is simply structurally more at risk for long distance spread due to its much lower population and farm density. For example, the average premises density in the high risk area identified in the Netherlands by Boender et al. [4] is over 4 premises/km^2^, while the average premises density in the high risk area in Minnesota given in Fig 5 is far less than 1 premises/km^2^. Similarly, as of 2016, the population density in the Netherlands is over 400 people/km^2^ [31], while the population density in Minnesota is about 25 people/km^2^ [32]. Such vast differences in the population and premises density could naturally lead to consistently longer distance transmission in Minnesota. Farm personnel, for example, would likely travel longer distances on average to work, and rendering trucks, identified as a risk factor in Iowa and Nebraska during the 2015 HPAI H5N2 outbreak [28] and in the Netherlands during the 2003 HPAI H7N7 outbreak [33], would likely travel longer distances to collect mortality from multiple premises in Minnesota than in Europe. These potential transmission pathways occurring over longer distances in Minnesota could result in an increased magnitude of *r*_0_.

Even though *r*_0_ is the only parameter estimated to be significantly different between the Minnesota, Netherlands, and Italy HPAI outbreaks, the size of *h*_0_ may be underestimated from the Minnesota outbreak based on the assumptions on infection status. Boender et al. [4] and Dorigatti et al. [6] assume the infectious period of a case premises ends with depopulation, whereas the assumption for the Minnesota kernel is that the infectious period ends following the start of carcass disposal, which leads to the longer average infectious period observed in the Minnesota cases: Based on the assumptions used to estimate the transmission kernel parameters, the average infectious period with 95% confidence interval was 7.47 (7.2, 7.8) days for cases in the Netherlands, 11.82 (6, 26) days for cases in Italy, and 17.22 (16.57, 17.87) days for cases in Minnesota [4, 6]. A longer infectious period would be expected to result in a proportionally smaller estimate for *h*_0_, as observed in the extended infectious period scenario compared to the baseline scenario in Table 1. Thus, the *h*_0_ estimate from the Minnesota outbreak would likely increase if the infectious period of a case premises were assumed to end after depopulation as in [4] and [6]. This issue highlights a limitation of the current study, the deterministic nature of the assumptions establishing the infectious status. As infection moving through a flock is subject to variability, the estimation of the transmission kernel could be improved by allowing for a range of time in which transitions in infection status could occur.

As it stands, though not statistically significant, the mean *h*_0_ estimate from Dorigatti et al. [6] and baseline Minnesota outbreak scenario are considerably higher than the estimate from Boender et al. [4]. The number of infected turkey premises as a proportion of the total number of cases was much higher in the Minnesota and Italy outbreaks than in the Netherlands outbreak [34, 35]. Turkeys are often observed to be more susceptible than chickens to avian influenza strains. For example, in the following experiments involving HPAI, turkeys were found to be more susceptible to HPAI H5N9 by Narayan et al. [36], and HPAI H7N1 and HPAI H5N1 by Aldous et al. [37]. Assuming turkeys were biologically more prone to infection during the outbreaks in the Netherlands, Italy, and Minnesota, the greater role of turkey premises in the estimation of the transmission kernels for the Minnesota and Italy outbreaks could have resulted in the larger mean estimates for *h*_0_.

Additionally, the industry practices in Italy at the time of the outbreak involving frequent equipment sharing, irregular application of basic biosecurity, and lack of physical barriers between poultry facilities, as cited by Capua et al. [38], could have contributed further to the larger magnitude of *h*_0_ estimated from the 2000 HPAI H7N1 outbreak. These industry practices would be expected to substantially increase the infection risk at close distances to an infectious premises, as observed in the Dorigatti et al. [6] transmission kernel.

Likewise, in Minnesota, the larger size of the premises could have contributed to the greater magnitude of the *h*_0_ estimate. Chis Ster et al. [39] and Boender et al. [40] included premises size in a transmission kernel model framework in an analysis of the 2001 FMD outbreak in Great Britain and 1997–1998 classical swine fever outbreak in the Netherlands, respectively. Both studies identified a non-linear relationship in which infectivity and susceptibility increase with premises size before reaching a saturation point after which any increase in the number of animals on a premises results in little increase in the premises-level infectivity and susceptibility. The applicability of these results to the poultry industry is uncertain, though Busani et al. [35] identified larger premises as having a higher risk of infection based on a Cox regression on data from the 2000 HPAI H7N1 outbreak in Italy (premises with more than 50,000 birds had a mean hazard ratio of 3.27 and 95% confidence interval of (2.25, 4.74) as compared to premises housing less than 10,000 birds). Therefore, the relatively higher *h*_0_ estimated from the Minnesota outbreak as compared to the estimate from the Netherlands outbreak could be attributed to differences in farm size, as premises in Minnesota house on average many more birds. However, further investigation into the relationship between farm size and infection risk during the Minnesota outbreak is needed.

The high risk area in Minnesota shown in Fig 5 contains the premises in Minnesota with basic reproduction numbers larger than one. These premises would be expected to on average infect at least one other premises, so an introduction into this area could result in sustained outbreak spread. Premises outside the high risk area of course can and do spread infection, but any chain of infections occurring outside the high risk area would be expected to die out relatively quickly. That 31% of the non-broiler premises were infected within the high risk area and 12% of the non-broiler premises were infected outside the high risk area in Minnesota suggests that extended chains of infection were indeed more pronounced within the high risk area. However, the results are complicated by the fact that several of the infections were likely primary introductions as evidenced by the statistical significance of the *k* parameter. It should be noted that *k* was not included in the estimation of the basic reproduction number since the risks captured by *k* were likely primarily from distance independent environmental pathways as opposed to pathways related to lateral spread.

The method used to estimate the basic reproduction number depends on the hazard rate estimated from the transmission kernel and the length of the infectious period of the premises assumed to be infected. Since the basic reproduction number is estimated using the transmission kernel, inter-premises distance plays a large role. As a result, premises centrality has a substantial impact on the basic reproduction number, as centrally located premises would pose a greater risk to a larger number of premises. The high risk area identified through this approach also relates to areas of high poultry density where a sustained outbreak could occur [4]. The similarity between the high risk area and areas with a high density of premises can be seen in a comparison of Fig 1 and Fig 5. The high risk area in Fig 5 appears smaller than the area with substantial premises density since the high risk area was estimated with broiler premises excluded, while the density plot was estimated using all premises types.

Based on the method used to estimate the basic reproduction number, any reduction in the hazard rate as given by the kernel (e.g., by implementing enhanced biosecurity), reduction in the infection period (e.g., by timely depopulation), or reduction in susceptible poultry flocks (e.g., by vaccination) would reduce the high risk area. Outbreak control strategies that are based on spatial transmission dynamics, such as a vaccination ring around an infectious premises, would likely have to be quite extensive to considerably reduce the high risk area in Minnesota since the transmission kernel estimates the hazard rate to be relatively high over moderate distances. Despite the apparent difficulty in reducing the risk of HPAI spread, Fig 6 provides evidence that the preemptive marketing implemented by poultry producers in a county in the high risk area during the outbreak in Minnesota could have decreased the potential for spread by reducing the susceptible population in the poultry-dense portion of the county. This result suggests that preemptive marketing could be a viable strategy to limit spread during an HPAI outbreak. On the other hand, there is also a risk of HPAI spread during movement due to the possibility of transporting infectious yet undetected birds [24]. Further work is required to more rigorously analyze the risks and benefits of sending poultry houses early to processing, and inform decisions related to prioritizing houses for early marketing and when these houses should be processed.
